# Epidemiological evaluation of Latvian control measures for African swine fever in wild boar on the basis of surveillance data

**DOI:** 10.1038/s41598-019-40962-3

**Published:** 2019-03-12

**Authors:** Katja Schulz, Edvīns Oļševskis, Christoph Staubach, Kristīne Lamberga, Mārtiņš Seržants, Svetlana Cvetkova, Franz Josef Conraths, Carola Sauter-Louis

**Affiliations:** 1 Friedrich-Loeffler-Institut, Federal Research Institute for Animal Health, Institute of Epidemiology, Südufer 10, 17493 Greifswald-Insel Riems, Germany; 2 Food and Veterinary Service, Riga, Peldu 30, LV-1050 Latvia; 3 Institute of Food Safety, Animal Health and Environment - “BIOR”, Riga, Lejupes 3, LV-1076 Latvia

## Abstract

A wild boar population infected with African Swine Fever (ASF) constitutes a constant threat to commercial pig farms and therefore to the economy of the affected country. Currently, ASF is still spreading in several countries and the implementation of intensive measures such as reducing wild boar population densities seems not to be able to stop the further spread of the disease. In addition, there are still substantial knowledge gaps regarding the epidemiology of the disease. To identify risk factors for a higher probability of a wild boar sample being virological or serological positive, comprehensive statistical analyses were performed based on Latvian surveillance data. Using a multivariable Bayesian regression model, the effects of implemented control measures on the proportion of hunted or found dead wild boar or on the estimated virus prevalence were evaluated. None of the control measures applied in Latvia showed a significant effect on the relevant target figure. Also, the estimated periodic prevalence of wild boar that had tested ASF positive by PCR appeared to remain unaffected over time. Therefore, there is an urgent need to reconsider the implemented control measures. The results of this study and the course of ASF in other affected countries, raise the question, whether an endemic situation of ASF in wild boar is reversible.

## Introduction

Due to its high case-fatality ratio, African Swine Fever (ASF) is one of the most dreaded viral diseases in swine, especially in countries with a considerable pig industry^1^. As yet, there is no effective treatment or vaccination available^2,3^.

ASF was introduced into South-Eastern Europe through Georgia. Shortly afterwards, the virus was detected in several other countries in the region^4–7^. It took seven years, until the epidemic had reached countries in the East of the European Union. Initially, Lithuania and Poland were affected, followed by Latvia and Estonia^6,8^. However, in the course of time, the disease also reached the Czech Republic, Romania, Hungary, Belgium and Bulgaria. As additional non EU countries, Moldova and since August 2018 also China are affected (OIE WAHID interface, visited online 13 August 2018). ASF has first been detected in the Latvian wild boar population in June 2014 and has been present there since then.

Outbreaks of ASF in domestic pigs occurred in several countries, but were usually controlled using conventional measures of animal disease control such as culling of affected pig holdings, safe disposal of the carcasses, cleaning and disinfection, movement restrictions, monitoring and surveillance. In regions, however, where ASF had the chance to spread in the wild boar population, controlling the disease in wild boar, not to mention eradicating it, was largely unsuccessful^4,9,10^. This is of particular importance as wild boar have been shown to play a significant role in disease transmission and maintenance^7,11^. Several strategies have been proposed to control ASF in wild boar population, including the ban of large-scale drive hunts, the implementation of massive targeted hunting and the removal and safe disposal of wild boar carcasses from the environment^12,13^. The effect of these measures is controversially discussed and many are assumed to have a low efficacy, e.g. use or ban of supplementary feeding of wild boar^8,14^. However, the restricting or prohibiting of drive hunts and paying incentives for carcass removal are considered to be reasonably effective^8,12^.

Nurmoja, *et al*.^9^ showed a positive association between wild boar population density and an increased incidence of ASF cases in wild boar. Accordingly, it is generally accepted that a drastic reduction of the wild boar population, at least in the surroundings of foci with the occurrence of ASF in wild boar may help to control ASF in this species^8,15^. Although this approach appears promising in the model, its practicability is doubtful. Lange^15^ showed by mathematical modelling that conventional strategies of wild boar population management (e.g. targeted hunting) must be conducted over several years to lead to a clear decrease of the wild boar population size. Furthermore, a recent study suggests that the role of the population density in the spread of ASF within wild boar might be less important than previously assumed^16^.

In Latvia, various control measures were implemented for different periods of time during the current epidemic (Table 1).

**Table 1:** Measures implemented to control African swine fever in wild boar in Latvia.

Measure	Control measure	Time period of measure implementation
A	Incentives to all persons who report dead wild boar to the veterinary authorities	July 2014–March 2015
B	Incentives to hunters (200 Euros per hunted wild boar older than 1 year, 50 Euros for wild boar of less than 1 year)	July 2014–Sept 2014
C	Collection and safe disposal of dead wild boar carcasses (done by the Food and Veterinary Service)	26 June 2014–March 2015
D	Notification, collection and safe disposal of dead wild boar carcasses (done by hunters)	April 2015–Jan2016
E	Collection and safe disposal of dead wild boar carcasses (Responsibility of local municipalities - mostly done by hunters)	Feb 2016–Dec 2017
F	Winter feeding ban	since 10 Dec 2014
G	Baiting of wild boar only allowed for hunting purposes	since 10 Dec 2014
H	Restrictions on driven hunts	Oct 2014–Feb 2015
		Oct 2015–Feb 2016
I	Incentives for hunting adult and sub-adult female wild boar	Nov 2015–March 2016
		Oct 2016–Dec 2017
J	Permission to use sound moderators (silencers) and night vision devices for wild boar hunting	since April 2015

However, the continuous spread of ASF in the wild boar population in Latvia suggests that these measures were not sufficient to contain the epidemic.

Following these observations, the present study aimed to evaluate the effects of the Latvian control measures on the respective target figures on the basis of available surveillance data, i.e. the effect on the proportion of samples originating from animals hunted or found dead or on the estimated ASFV genome prevalence.

Due to the existing knowledge gaps regarding the epidemiology of ASF, risk factors for a higher probability to detect ASF positive samples were additionally identified.

The results of the study may be used to improve assessments of the success of control measures for ASF in wild boar. They also allow adjusting the applied measures as appropriate.

## Material and Methods

### Study area

**Figure 1:**
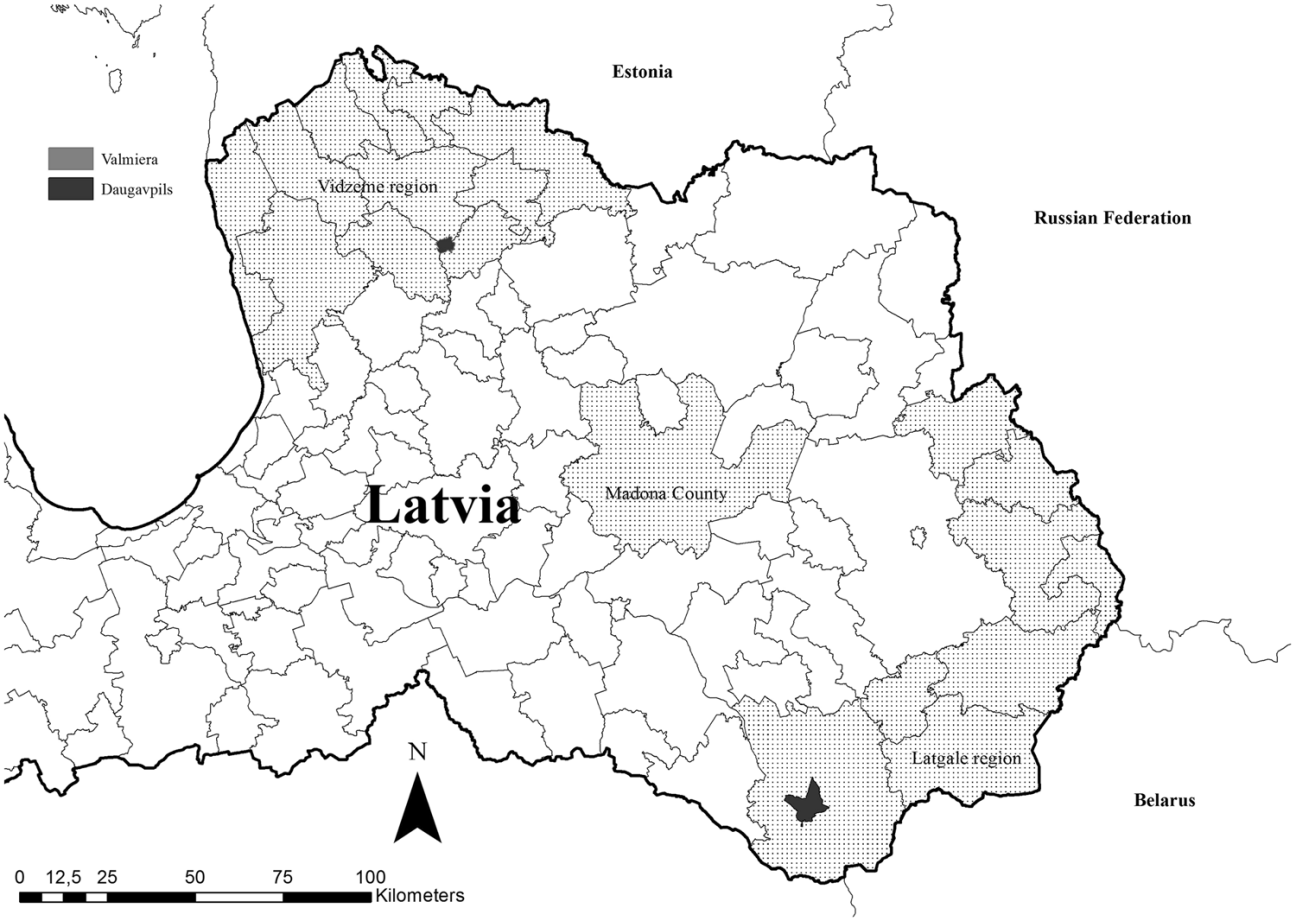
The study area comprised of three regions, i.e. Latgale region in the South, Vidzeme region in the North and Madona County in between. Map was generated by using ArcGIS ArcMap 10.3.1 (ESRI, Redlands, CA, USA).

The study area consisted of three different regions in Latvia, from where a sufficiently large sample size was available, i.e. Latgale region, which is bordering Belarus, Vidzeme region, bordering Estonia, and Madona County, which is located between the two regions (Fig. 1). In total, the study area included 20 counties and comprised of approximately 15,146 km². The towns of Daugavpils and Valmiera were excluded from the analyses.

### Data

Surveillance data were extracted from the CSF/ASF wild boar surveillance database of the European Union (https://surv-wildboar.eu). The study period ranged from 25.06.2014 (first detection of ASF in wild boar in Latvia) to 31.10.2017 (41 months). There was one individual data set for each wild boar, which contained information about date and location where the sampled animal had been hunted or found dead, the estimated age of the animal and the laboratory test result of the sample (detection of virus genome by PCR and serology). Regarding age, animals were categorized in two classes, younger than one year and one year or older. Furthermore, information was recorded for the origin of each sample (in the following text termed carcass type), i. e. shot apparently healthy (active surveillance) or shot sick, found dead or involved in a road traffic accident (passive surveillance). Data with an inconclusive result in any of the variables were excluded.

Laboratory testing of samples for ASFV genome and ASF-specific antibodies was performed in the Latvian Institute of Food Safety, Animal Health and Environment (BIOR) by the Animal Disease Diagnostic Laboratory. For antibody detection and ASFV genome, blood samples were taken by the hunters from hunted wild boar. Organ (kidney, spleen, lymph node) or bone marrow samples were collected from animals found dead and analyzed for virus genome.

In 2014 and 2015, detection of ASFV genome was performed by real time PCR^17^, but from 2016 onwards, the UPL protocol established by Reference Laboratory for ASF of the European Union^18^ was used. Antibody testing was done by using a commercial blocking enzyme-linked immunosorbent assay (ELISA) (INGENASA, INGEZIM PPA COMPAC, Spain) and positive results confirmed with the indirect immunoperoxidase test (IPT), validated by the Reference Laboratory for ASF of the European Union.

The State Forest Service provided wild boar population data that were used to assess the wild boar population density. Data were available on game management unit for the hunting seasons (April of a year until March of the following year) during the ASF epidemic (2014/15, 2015/16, 2016/17). The definition of the game management units is based on habitat and human related landscape characteristics (e.g. human population density, land use, major roads and rivers). They are used to manage all species of game. The number of wild boar in each game management unit was estimated on the basis of the hunting bag, population structure data (number of hunted animals divided by their age and gender), current occurrence evidences (e.g. visual observations, foot prints etc. observed by hunters or forest rangers) and information on wild boar damage to crop production claimed by local farmers. Estimations were annually done with a deadline on the 1st of April of each year for the previous year, for each game management unit by a local official of the State Forest Service, who collected and amalgamated the information.

For analysis, the data was aggregated at municipality level. The software ArcGIS ArcMap 10.3.1 (ESRI, Redlands, CA, USA (http://www.esri.com/) was used to calculate the wild boar density per km² based on the estimated number of wild boar per game management unit. Areas of game management units that overlapped with the territories of at least two municipalities were proportionally attributed to the territory of each municipality. On the basis of the wild boar density per km² and the adapted game management units, the total number of wild boar per municipality was calculated. The average values of the wild boar densities of the three hunting seasons were determined and assigned to each corresponding municipality. An additional dataset on wild boar population density was available from the time before ASF had emerged in Latvia (hunting season 2011/12). In contrast to the hunting data of the years during the ASF epidemic, these data were only available on regional subunits of the State forest service (geographically larger than municipalities). These data were assigned to the administrative district level. To ensure comparability, the data of the hunting seasons during the ASF epidemic had to be adapted accordingly. To this end, the centroid of the municipalities was used to assign the wild boar densities of the municipalities to the respective administrative district. The number of wild boar in the individual municipalities, which belonged to one particular administrative level, were summarized and the wild boar densities for each administrative district calculated.

### Maps and figures

Maps were generated using ArcGIS ArcMap 10.3.1 (ESRI, Redlands, CA, USA, http://www.esri.com/). Figures were generated using the software package R (http://www.r-project.org)^19^.

### Statistical analyses

Statistical analyses were performed using the software package R (http://www.r-project.org) if not stated otherwise. Confidence intervals were calculated according to Clopper and Pearson^20^. A p-value of ≤0.05 was considered statistically significant.

The statistical analyses of population data and the association between potential risk factors for a higher probability to detect ASF-positive samples and the ASF laboratory test result were performed essentially as described by Nurmoja, *et al*.^9^. Correspondingly, potential associations between age and test result as well as carcass type and test result were examined using Fisher’s exact test. Potential associations between carcass type and age were analyzed in the same manner. The estimated periodic prevalence of ASFV genome-positive wild boar and the seroprevalence of ASF-specific antibodies with the respective 95% confidence intervals were calculated and the temporal course of the prevalence data was analyzed.

Potential differences in population densities between different hunting seasons were investigated using a non-parametric Kruskal-Wallis test. If a statistically significant difference was detected, pairwise Mann–Whitney U tests were performed. To control the type I error for multiple testing, the Bonferroni correction^21^ was applied in these comparisons.

**Figure 2:**
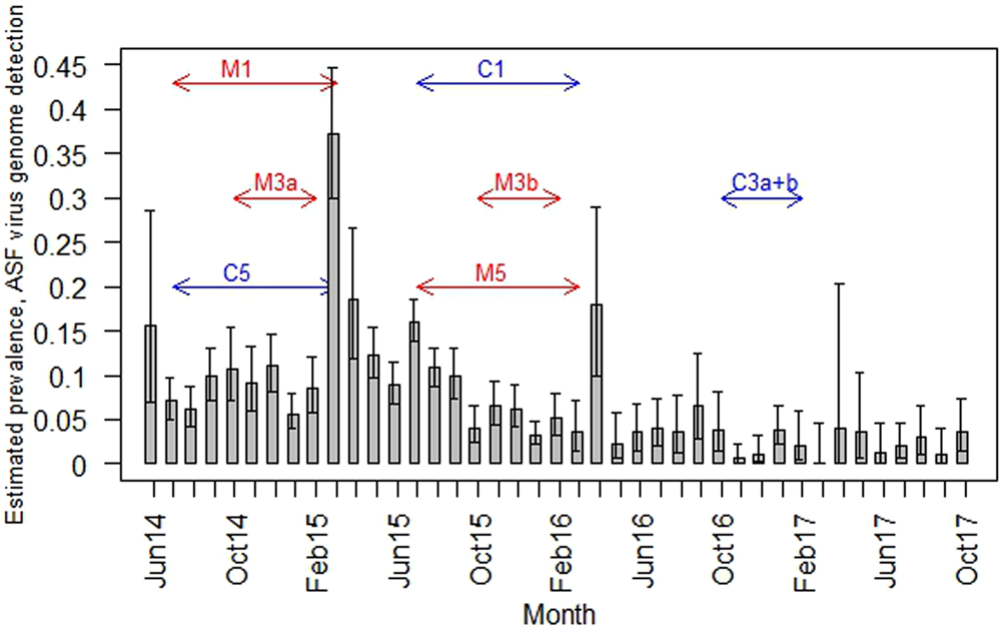
Estimated prevalence of African Swine Fever virus genome-positive wild boar (boxes) and 95% confidence intervals (whiskers) for each month of the study period. Red arrows illustrate the time span for the different measures, which aimed to influence the prevalence. Blue arrows illustrate the corresponding control periods. The numbering of measures and controls follows Table 2. Figure was generated by using the software package R (http://www.r-project.org).

**Figure 3:**
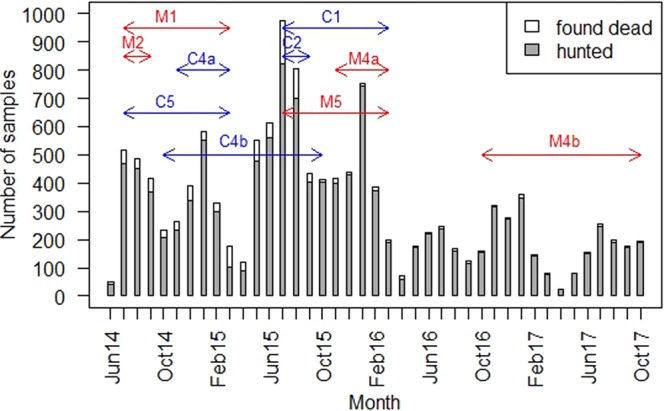
Number of samples originating from hunted wild boar or wild boar found dead. Red arrows illustrate the periods, when various measures that aimed at influencing the proportion of hunted/found dead wild boar were applied. Blue arrows illustrate the corresponding control periods. The numbering of measures and controls was performed according to Table 2. Figure was generated by using the software package R (http://www.r-project.org).

To study the effect of control measures on the wild boar population, or the estimated periodic ASFV genome prevalence, an appropriate control period was defined for each period, during which the specific measure was in place (Table 2, Figs 2 and 3). When determining the control period, we aimed to choose the same number of month and the same season as in the period, during which the measure was applied. Ideally, no other measure with a potential impact on related parameters (e.g. increase of hunted wild boar/increase of animals found dead) was in place during the control period. Accordingly, for some measures the control periods had to be chosen before, and for others after the periods, when the measures were applied.

**Table 2:** Control measures for African swine fever in wild boar in Latvia analyzed for their effect on the estimated ASFV genome prevalence, the proportion of hunted animals or found dead.

	Measure	Measure/Control period
		Null hypothesis
M1	Incentives to all persons who report dead wild boar to the veterinary authorities (corresponds to A in Table 1)	Measure period (M1 = C5): July 2014 – March 2015 Control period (C1 = M5): July 2015 – March 2016
		H_0_1: No significant effect of M1on the **proportion of animals found dead** H_0_2: No significant effect of M1on the **estimated ASFV genome prevalence**
M2	Incentives to hunters for hunted wild boar (corresponds to B in Table 1)	M2: July 2014 – Sept 2014 C2: July 2015 – Sept 2015
		H_0_: No significant effect of M2 on **the proportion of hunted animals**
M3	Restrictions on driven hunts (corresponds to H in Table 1)	M3a: October 2014 – February 2015 M3b: October 2015 – February 2016 C3a + b: October 2016 – February 2017
		H_0_: No significant effect of M3 on the **estimated ASFV genome prevalence**
M4	Incentives for hunting adult and sub-adult female wild boar (corresponds to I in Table 1)	M4a: November 2015 – March 2016 M4b: October 2016 – October 2017 C4a: November 2014 – March 2015 C4b: October 2014 – October 2015
		H_0_: No significant effect of M4 on **the proportion of hunted animals**
M5	Permission to use sound moderators (silencers) and night vision devices for wild boar hunting (corresponds to J in Table 1)	M5 = C1: July 2015 – March 2016 C5 = M1: July 2014 – March 2015
		H_0_1: No significant effect of M5 on **the proportion of hunted animals** H_0_2: No significant effect of M5 on the **estimated ASFV genome**

For some measures, no control period could be established. These measures were excluded from the analysis (measures F and G in Table 1). Moreover, only measures with a direct effect on the composition of the population, the carcass type (animals hunted or found dead) or the estimated ASFV genome prevalence were considered. Accordingly, measures C, D and E (Table 1) were excluded from further analysis.

To test the Null hypotheses and thus the effect of the measure on the estimated ASFV genome prevalence or the proportion of hunted/found dead wild boar a multivariable Bayesian regression model was used^9,22^. A proportion of mean/Std.Dev. >1.96 was regarded as statistically significant.

Each measure was defined as the independent variable. In each model, age was included as fixed effect. Accounting for the epidemiological situation over time, in the case of carcass type (proportion of hunted/found dead wild boar) as dependent variable, the estimated ASFV genome prevalence in each month of the respective measure/control period was included as fixed effect. The origin of sample (hunted/found dead) was included as fixed effect in the models where the estimated ASFV genome prevalence constituted the dependent variable. Time (in months) was treated as a random effect. To estimate the parameter values, a Markov Chain Monte Carlo algorithm (MCMC) was used. 50,000 iterations were performed and at every 50th iteration a sample was selected. For the burn-in 1,000 iterations were chosen. The model was implemented using BayesX 2.0.1 (http://www.uni-goettingen.de/de/bayesx/550513.html) and convergence of the MCMC chain was assessed using standard diagnostic plots and tests. A detailed model description can be found in the supplementary information.

Stepwise model building using forward selection were used to find the best model for each measure. The models were evaluated on the basis of their Deviance Information Criterion (DIC) and the Effective number of parameters (pD). For each measure the model with the smallest DIC were used for the final analyses. In the case of comparable DICs, the model with the smallest pD was chosen (Supplementary information Tables S2, S8).

## Results

### Data

In total, 12,978 data records were available for analysis. Of these, 5,224 records originated from Latgale region, 5,379 from Vidzeme region and 2,375 from Madona County.

### Statistical analysis

A significant association between age and positive laboratory test results (both, ASFV genome and serology) was found. When analyzing all samples, the probability to find a positive test result was higher in young animals (Table 3). The probability that a sample originating from an animal found dead came from a younger animal was significantly higher as compared to older wild boar. The probability to detect a positive test results in wild boar found dead was significantly higher than in hunted animals (Table 3).

**Table 3:** Associations between potential risk factors (age and carcass type) and a positive laboratory test result (ASFV genome detection and serology) and association between age and carcass type.

Age	ASFV genome detection	p	<0.001
		OR (95% CI)	0.55 (0.47–0.64)
	Serology	p	<0.001
		OR (95% CI)	0.72 (0.60–0.87)
	Carcass type (hunted/found dead)	p	<0.001
		OR (95% CI)	0.70 (0.60–0.82)
Carcass type (hunted/found dead)	ASFV genome detection	p	<0.001
		OR (95% CI)	192.51 (157.37–235.49)
	Serology	p	<0.001
		OR (95% CI)	9.30 (6.83–12.67)

**Figure 4:**
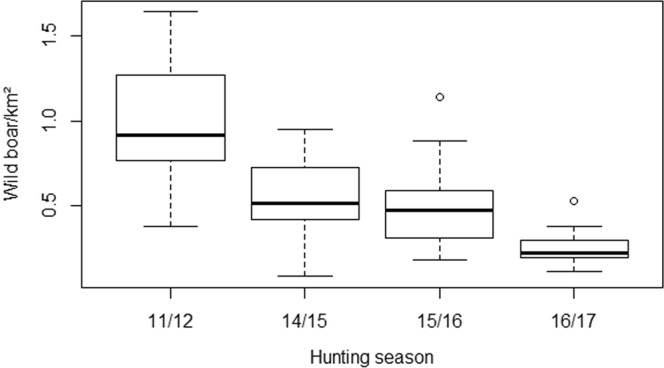
Temporal course of wild boar population density. Figure was generated by using the software package R (http://www.r-project.org).

Temporal changes in population density were also detected: Compared to the hunting season of 2011/12, the wild boar density decreased significantly starting with 2014 until the hunting season of 2016/17 (p < 0.001) (Fig. 4).

**Figure 5:**
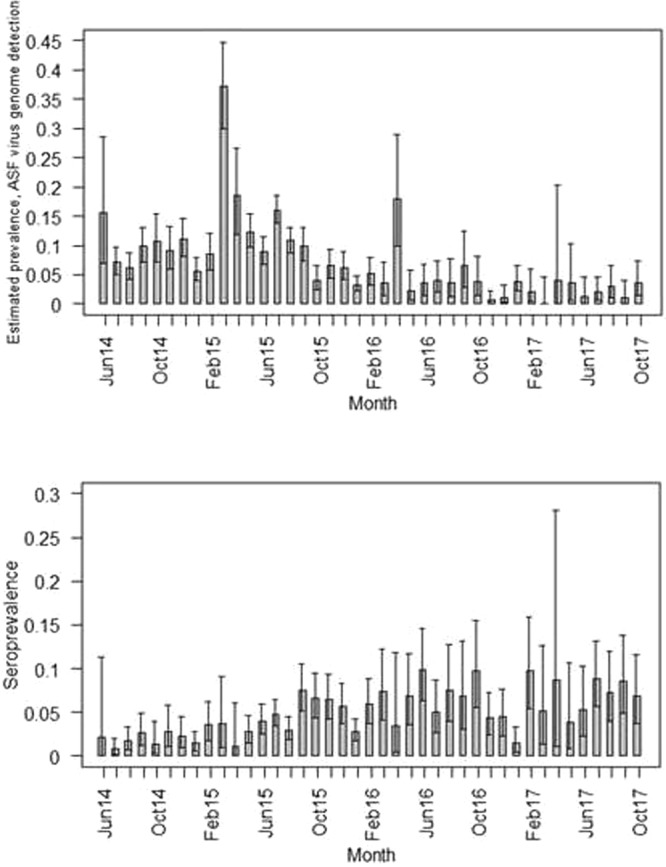
Estimated ASF prevalence (upper panel ASF virus genome detection by PCR; lower panel, detection of ASF-specific antibodies). The boxes represent point estimates and the whiskers 95% confidence intervals. Figure was generated by using the software package R (http://www.r-project.org).

The estimated ASF prevalence, regardless of the laboratory testing method, i.e. ASFV genome detection or serology, hardly showed any statistically significant changes during the study period of 41 months (Fig. 5).

For the model analyses, models with the lowest DIC were used (Table 4, Supplementary information Tables S2, S8). None of the measures resulted in a significant effect on the variable of interest (carcass type or estimated ASFV genome prevalence).

**Table 4:** Results of the multivariable analyses regarding the effects of the control measures on the estimated ASFV genome prevalence, the proportion of hunted animals or found dead.

Measure	Null hypotheses	Mean/St.Dev.^*^
Incentives to all persons who report dead wild boar to the veterinary authorities (M1)	H_0_1 No effect on the proportion of animals found dead	0.72
	H_0_2 No effect on the estimated virus prevalence	1.20
Incentives to hunters for hunted wild boar (M2)	H_0_ No effect on the proportion of hunted animals	0.65
Restrictions on driven hunts (M3a)	H_0_ No effect on the estimated virus prevalence	0.61
Restrictions on driven hunts (M3b)	H_0_ No effect on the estimated virus prevalence	0.58
Incentives for hunting adult and sub-adult female wild boar (M4a)	H_0_ No effect on the proportion of hunted animals	0.09
Incentives for hunting adult and sub-adult female wild boar (M4b)	H_0_ No effect on the proportion of hunted animals	0.78
Permission to use sound moderators (silencers) and night vision devices for wild boar hunting (M5)	H_0_1 No effect on the proportion of hunted animals	1.71
	H_0_2 No effect on the estimated virus prevalence	1.30

^*^Mean/Std.Dev. **>**1.96, indicating statistical significance.

Paying incentives to all persons who reported dead wild boar to the veterinary authorities (control measure M1, Table 2) yielded a greater effect on the estimated ASFV genome prevalence (1.20) than most of the other measures. However, the difference between the estimated prevalences in the two time periods was nearly zero (Supplementary information Table S1). Also, permission to use silencers and night vision devices for wild boar hunting (control measure M5) seemed to have a stronger effect, albeit not significant, on the proportion of hunted wild boar and on the estimated ASFV genome prevalence (Table 4). In addition, the number of hunted wild boar was larger in the period when control measure M5 had been implemented (Supplementary information Table S1).

Paying incentives to hunters for hunted wild boar (control measure M2) had no statistically significant effect on the proportion of hunted animals (Table 4) but also a lower number of hunted animals in the time when the measure was applied as compared to the time, when it was not applied (Supplementary information Table S1).

The estimated ASFV genome prevalence was higher in the period when driven hunts were restricted (control measures M3a and M3b) as compared to the time, when there were no limitations of driven hunts (Supplementary information Table S1). These finding was also supported by the model analyses. However, the effect of the measure on the estimated ASFV genome prevalence was not significant (Table 4). Paying incentives for hunting adult and sub-adult female wild boar (control measure M4a and M4b) also failed to show a statistically significant effect on the proportion of hunted wild boar in any of the periods, during which this control measure was applied (Table 4).

## Discussion

The aim of the study was to evaluate the effect of measures that had been applied in Latvia to control ASF in wild boar. In view of the fact that there are still substantial gaps in our knowledge about the epidemiology of ASF, particularly in wild boar^23,24^, we also tried to address some of these gaps. Therefore, risk factors for a higher probability to detect ASF-positive wild boar were investigated.

In close cooperation with the Latvian veterinary authorities, three regions were determined as the study area. From these regions, which were typical for other parts of the country, also with regard to the occurrence of ASF in wild boar, a wide range of surveillance and wild boar population data originating from different epidemiological situations were available.

According to EU legislation (Council Directive 2002/60/EC), it is mandatory to sample all hunted and dead wild boar in ASF-affected areas. It can thus be assumed that the obtained sample size in areas affected by ASF corresponds to the entire hunting bag, i.e. usually at least 60–80% of the population. However, due to hunting habits such as the desire for trophies, some older and very young wild boar are usually not shot. Therefore, the composition of the hunting bag does not completely mirror the structure of the wild boar population^25–28^. Nonetheless, the sample size available from ASF- affected areas is still large as compared to other wildlife studies^29–32^. Consequently, although respecting the uncertainty regarding the true population size and the true epidemiological situation of ASF in Latvian wild boar, we assumed that the sample size was sufficient for our analyses to estimate the periodic ASFV genome prevalences and seroprevalences with a high confidence.

The results regarding the association of risk factors and laboratory test results were in line with results obtained in a study recently conducted in Estonia^9^. The majority of samples resulted from adult wild boar and was obtained by active surveillance. However, the probability to find an ASF-positive animal was significantly higher in wild boar found dead, i.e. passive surveillance. This result is in accord with previous findings and emphasizes the urgent need to enhance the number of samples resulting from passive surveillance^7,9,33–35^. The fact that similar results were obtained in several countries affected by ASF in wild boar suggests that the risk factors for a higher probability to detect an ASF-positive sample are also similar, regardless of the environment, the region or the population density. Therefore, one can assume that the course of ASF within wild boar populations is comparable between affected countries or regions. The increased probability that a wild boar that was found dead was younger than one year suggests that young animals are at a higher risk to die. The cause of death may be ASF in the study area, but other reasons such as infections with agents other than ASFV or predation cannot be excluded^28,36^.

Although estimated population densities in different hunting seasons may not be independent, due to the lack of a suitable alternative, independence was assumed and the conservative non-parametric Kruskal-Wallis test used for statistical comparison.

Over the years, a significant reduction of the estimated wild boar population density was observed. This is probably due to the continuous spread of ASF and its high case-fatality ratio. To rule out completely that the decrease was not just an effect of intensified hunting in these areas, further data, from the western part of Latvia should be analyzed and compared. In these regions, ASF cases occurred after 2015 but hunting strategies were the same as in affected regions. However, such analyses were beyond the scope of the present study as only data of the defined study area were used. Moreover, only data of one hunting season (2011/12) before the emergence of ASF were available. Therefore, it remains unknown, if the population density in the hunting years 2012/13 and 2013/14 was higher than in the years after ASF had been introduced into the wild boar population. Furthermore, when interpreting the temporal trend of the wild boar population density, it has to be kept in mind that data were available on different administrative levels, which may have caused some bias. Since population density was estimated with some degree of uncertainty, the effect of the bias caused by aggregating data originally obtained on different administrative levels is likely to be negligible. As wild boar population densities usually represent estimates, these data have always to be regarded as benchmarks rather than precise figures. However, currently these rough estimates of population densities based on hunting bag etc. are the only data available for analyzing wild boar population density data.

Due to the inclusion of all available samples in the analyses, the estimated periodic prevalences could be determined. The analyses of estimated ASFV genome prevalences during the study period showed hardly any change during the study period. This finding is in accord with other studies where the course of ASF in wild boar was analyzed and no significant change in the ASF prevalence in wild boar could be found over time^9,35^. The small variation in the prevalences did not indicate any seasonality of the ASF infection in wild boar. This is in contrast to studies conducted in the Russian Federation, Poland and Sardinia, where seasonality in the occurrence of ASF was found^35,37,38^.

Only some of the control measures that had been implemented by the Latvian authorities during the study period could be analyzed with regard to their effect on the course of ASF. For measures that started shortly after the first ASF-positive wild boar had been detected in Latvia and were in place during the entire study period, e.g. the winter feeding ban (Table 1), an appropriate control time period could not be defined. Due to lack of transferability to other countries or areas, measures, in which only the allocation of responsibilities (e.g. veterinary authorities were responsible for collection of dead wild boar vs. hunters were responsible; C, D and E in Table 1) changed, were excluded from the analyses.

To analyze the effects of different control measures on the carcass type (hunted wild boar, animals found dead) or the estimated ASFV genome prevalence, a multivariable Bayesian regression model was used. Age and carcass type were statistically significantly associated with the estimated ASFV genome prevalence (Table 3). Therefore, accounting for the effect of age and either carcass type or estimated ASFV genome prevalence on the dependent variable, these variables were included in the model as fixed effects. Due to the low number of available geographical units, no spatial effect was included.

In contrast to most control measures, paying incentives to all persons who report dead wild boar to the veterinary authorities (control measure M1) and permission to use silencers and night vision devices for wild boar hunting (control measure M5) indicated a potential slight effect on the estimated ASF prevalence. Moreover, the number of hunted wild boar increased in the period, when control measure M5 was applied. The analyses of the effect of control measure M1 (incentives for notification of found dead wild boar) could not be separated from the analyses of control measure M5 (hunters could use silencers and night-vision device for wild boar hunting), because the control period of M1 corresponded to the period, when control measure M5 was applied. Therefore, the potential effect of M1 may be a result of M5 or vice versa. In addition, the combination of these measures may have also led to the observed results. The available data suggest that the wild boar population density decreased over time, presumably due to the increased hunting pressure which might have been supported by control measure M5 (usage of supporting tools). These results demonstrate the need to adapt hunting regulations in the case of the emergence of ASF or even before as a preventive measure in an area at risk. The permission to use these tools, however, does not necessarily indicate the extent of their use by hunters.

Paying incentives for hunted wild boar (M2) in the summer months of 2014 was mainly done to facilitate hunting. The season for driven hunts starts in October and the number of hunted animals therefore usually increases from this month onwards without any additional measures. Measure M2 showed no effect in the present study However, motivating hunters financially to reduce the population density might still support the control of ASF, as the wild boar population density probably plays an important role in the spread of ASF^8,9,15^. This applies especially to the summer months, during which the hunting activity is usually decreased.

As driven hunts end in February, restrictions regarding this form of hunting were only applied until February 2015 (M3a) and in a second period until February 2016 (M3b). The restrictions of driven hunts had no significant effect on the estimated ASFV genome prevalence. In both time periods when control measure M3 was implemented, the estimated ASFV genome prevalence was even higher than in the respective control periods. These findings contradict the hypothesis that driven hunts increase the risk of spreading ASF, as infected wild boar might be disturbed, driven apart and stimulated to move in a wider radius as usual.

Control measures 4a and 4b consisted of paying incentives for hunted female wild boar. Due to ethical considerations (hunting restrictions during the time of wild boar reproduction), no incentives were paid from March 2016 to September 2016. The implementation of control measure M4a led to an increase of the number of hunted wild boar, but the effect of the measure was not statistically significant, when age and prevalence were taken into account. When control measure 4b was in place, the incentives were lower than during the period, when control measure 4a was applied. This was simply to reduce expenses as limited funds were available. Although control measure 2, which was implemented in summer 2014, already included paying incentives for hunted wild boar, the payments only started again in November 2015. At that time, it had become obvious that ASF continued to spread. Accordingly, the European Commission decided to provide Latvia financial support to reduce the wild boar population more intensively. Statistical analysis showed that similar to 2014, paying incentives had a direct positive effect on the proportion of hunted wild boar.

None of the control measures applied in Latvia during the present ASF epidemic showed a statistically significant effect on the relevant target variable (increase of the number of hunted wild boar or wild boar found dead). Also, no significant effect on the estimated ASF prevalence could be observed. However, when interpreting the results, the partly low sample size has to be considered. Especially the samples showing a positive test result for ASFV were often very small (Supplementary information Table S1) and a decrease of the effect size due to a small sample size could therefore not be excluded.

Over the study period of 41 months, the prevalence failed to decrease significantly, irrespectively of the implemented measures. Therefore, analyzing surveillance data, this study demonstrates what many experts had already feared and what also became evident in other affected countries: Once ASF has emerged in the wild boar population in a region, it seems hard or even impossible to eradicate the disease, at least if the epidemic is not stopped very early on. However, any potential long-term effects of the control measures remain unknown. A minor or moderate decrease of the ASF incidence in the wild boar population over several years due to the implemented control measures is still possible. Also Lange^15^ stated that an effect on the course of ASF and the population will probably take many years. However, due to available data, in the present study the effects were only investigated for the measure and the defined control period.

Recent analyses of unpublished surveillance data suggest that the proportion of wild boar samples that tested positive for antibodies to ASFV but negative for ASFV genome by PCR increases in Latvia. This may indicate a decrease in the incidence of ASF in wild boar^39,40^.

Our findings also demonstrate that more detailed knowledge on the transmission and excretion of ASFV, the tenacity in wild boar carcasses and on the role of potential carriers is necessary^40^. Once the epidemiology of the disease in the wild boar population becomes clearer, it might be possible to improve control measures and to utilize them in a more targeted way. Also, alternative measures such as fencing, which were used in controlling an epidemic of ASF among wild boar in the Czech Republic, need to be considered. Further studies are required to verify the success of such alternative control measures but also to investigate the possible long-term effects of measures that were already applied.

Although the effect of the wild boar population density on the course of an ASF epidemic remains disputed^8,9,16^, additional strategies to reduce the wild boar population density should be taken into account (e.g. intensive targeted hunting on adult females or trapping). However, finally the question remains, whether affected countries will have to accept the presence of ASF within their wild boar population at a certain level and how they and their trade partners can learn to live with this situation. It may therefore be inevitable to focus on the biosecurity of pig farms. Unaffected countries should carefully evaluate their surveillance with the aim of preventing ASF entry and early detection in the case of ASF introduction^41^.

## Supplementary information

Supplementary information

## Data Availability

In accordance with the responsible persons from Latvia, the original data used for the analyses can be obtained from the author.

## References

[1] Penrith ML, Vosloo W (2009). Review of African swine fever: transmission, spread and control. Journal of the South African Veterinary Association-Tydskrif Van Die Suid-Afrikaanse Veterinere Vereniging.

[2] Rock DL (2017). Challenges for African swine fever vaccine development-“… perhaps the end of the beginning”. Vet Microbiol.

[3] Zakaryan H, Revilla Y (2016). African swine fever virus: current state and future perspectives in vaccine and antiviral research. Vet Microbiol.

[4] Gogin A, Gerasimov V, Malogolovkin A, Kolbasov D (2013). African swine fever in the North Caucasus region and the Russian Federation in years 2007–2012. Virus Research.

[5] Wozniakowski G (2016). Current status of African swine fever virus in a population of wild boar in eastern Poland (2014–2015). Arch Virol.

[6] Sanchez-Vizcaino JM, Mur L, Gomez-Villamandos JC, Carrasco L (2015). An Update on the Epidemiology and Pathology of African Swine Fever. J comp Pathol.

[7] Oļševskis E (2016). African swine fever virus introduction into the EU in 2014: Experience of Latvia. ResVet Sci.

[8] Gavier-Widen D (2015). African swine fever in wild boar in Europe: a notable challenge. Vet Rec.

[9] Nurmoja I (2017). Development of African swine fever epidemic among wild boar in Estonia - two different areas in the epidemiological focus. Sci Rep.

[10] Depner K (2016). African Swine fever - epidemiological considerations and consequences for disease control. Tieraerztliche Umschau.

[11] Pejsak Z, Truszczynski M, Niemczuk K, Kozak E, Markowska-Daniel I (2014). Epidemiology of African Swine Fever in Poland since the detection of the first case. Polish Journal of Veterinary Sciences.

[12] Guinat C (2016). Effectiveness and practicality of control strategies for African swine fever: what do we really know?. Vet Rec.

[13] European Food And Safety Authority. Scientific opinion on African swine fever. *EFSA J* **13**(**7**), 4163, 10.2903/j.efsa.2015.4163 (2015). 10.2903/j.efsa.2015.4163 PMC13151619 42109810

[14] Geisser H, Reyer HU (2004). Efficacy of hunting, feeding, and fencing to reduce crop damage by wild boars. Journal of Wildlife Management.

[15] Lange, M. Alternative control strategies against ASF in wild boar populations. *EFSA supporting publication* **EN-843** (2015).

[16] Chenais E, Ståhl K, Guberti V, Depner K (2018). Identification of Wild Boar–Habitat Epidemiologic Cycle in African Swine Fever Epizootic. Emerging Infectious Diseases.

[17] King DP (2003). Development of a TaqMan (R) PCR assay with internal amplification control for the detection of African swine fever virus. Journal of Virological Methods.

[18] Fernandez-Pinero J (2013). Molecular Diagnosis of African Swine Fever by a New Real-Time PCR Using Universal Probe Library. Transboundary and Emerging Diseases.

[19] R Core Team. R: A Language and Environment for Statistical Computing. *R Foundation for Statistical Computing*, *Vienna*, *Austria* (2015).

[20] Clopper CJ, Pearson ES (1935). The use of confidence or fiducial limits illustrated in the case of the binomial. Biometrika.

[21] Dunn OJ (1961). Multiple comparisons among means. Journal of the American Statistical Association.

[22] Staubach C (2011). Bayesian space-time analysis of Echinococcus multilocularis-infections in foxes. Veterinary Parasitology.

[23] Sanchez-Cordon PJ, Montoya M, Reis AL, Dixon LK (2018). African swine fever: A re-emerging viral disease threatening the global pig industry. Veterinary Journal.

[24] Schulz, K., Staubach, C. & Blome, S. African and classical swine fever: similarities, differences and epidemiological consequences. *Veterinary Research* **48**, 10.1186/s13567-017-0490-x (2017). 10.1186/s13567-017-0490-x PMC5706370 29183365

[25] Stubbe W, Stubbe M (1977). Vergleichende Beiträge zur Geburts- und Reproduktionsbiologie von Wild- und Hausschwein-Sus scrofa L., 1758. Beiträge zur Jagd- und Wildforschung.

[26] European Food And Safety Authority. Control and eradication of Classic Swine Fever in wild boar and Animal health safety of fresh meat derived from pigs vaccinated against Classic Swine Fever. *The EFSA Journal* **932** (2008).

[27] Rossi S (2005). Incidence and persistence of classical swine fever in free-ranging wild boar (Sus scrofa). Epidemiology and Infection.

[28] Keuling O (2013). Mortality rates of wild boar Sus scrofa L. in central Europe. European Journal of Wildlife Research.

[29] Adamovicz, L. *et al*. Investigation of multiple mortality events in eastern box turtles (Terrapene carolina carolina). *Plos One* **13**, 10.1371/journal.pone.0195617 (2018). 10.1371/journal.pone.0195617 PMC5886585 29621347

[30] Baker, R., Scott, D. M., Keeling, C. & Dwight, C. Overwinter survival and post-release movements of translocated water voles: implications for current mitigation guidance. *European Journal of Wildlife Research* **64**, 10.1007/s10344-018-1216-8 (2018).

[31] Burkholder EN, Jakes AF, Jones PF, Hebblewhite M, Bishop CJ (2018). To Jump or Not to Jump: Mule Deer and White-Tailed Deer Fence Crossing Decisions. Wildlife Society Bulletin.

[32] Walton L (2016). The ecology of wildlife disease surveillance: demographic and prevalence fluctuations undermine surveillance. Journal of Applied Ecology.

[33] Schulz K, Calba C, Peyre M, Staubach C, Conraths FJ (2016). Hunters’ acceptability of the surveillance system and alternative surveillance strategies for classical swine fever in wild boar - a participatory approach. BMC Veterinary Research.

[34] European Food Safety Authority, Cortinas Abrahantes, J., Gogin, A., Richardson, J. & Gervelmeyer, A. Scientific report on epidemiological analyses on African swinefever in the Baltic countries and Poland. *EFSA J* **15**(**3**), 4732 (2017). 10.2903/j.efsa.2017.4732 PMC7010137 32625438

[35] Smietanka K (2016). African Swine Fever Epidemic, Poland, 2014–2015. Emerging Infectious Diseases.

[36] Briedermann, L. *Schwarzwild*. (Dt. Landwirtschaftsverl. 1990).

[37] Iglesias I, Rodriguez A, Feliziani F, Rolesu S, de la Torre A (2017). Spatio-temporal Analysis of African Swine Fever in Sardinia (2012–2014): Trends in Domestic Pigs and Wild Boar. Transboundary and Emerging Diseases.

[38] Khomenko, S. *et al*. African Swine Fever in the Russian Federation: Risk Factors for Europe and Beyond. *EMPRES Watch*, 28 (2013).

[39] Zani, L. *et al*. Deletion at the 5′-end of Estonian ASFV strains associated with an attenuated phenotype. *Sci Rep* **8**, 10.1038/s41598-018-24740-1 (2018). 10.1038/s41598-018-24740-1 PMC5916933 29695831

[40] Petrov A, Forth JH, Zani L, Beer M, Blome S (2018). No evidence for long‐term carrier status of pigs after African swine fever virus infection. Transboundary and Emerging Diseases.

[41] Schulz, K. *et al*. Surveillance strategies for Classical Swine Fever in wild boar – a comprehensive evaluation study to ensure powerful surveillance. *Sci Rep* **7**, 43871, 10.1038/srep43871https://www.nature.com/articles/srep43871#supplementary-information (2017). 10.1038/srep43871 PMC5339697 28266576

