# Supplementary material for: Epidemiological evaluation of Latvian control measures for African swine fever in wild boar on the basis of surveillance data
Source: Sci Rep. 2019 Mar 12;9:4189. doi: 10.1038/s41598-019-40962-3 (PMC6414528; doi:10.1038/s41598-019-40962-3)
Supplement: Supplementary file 1 — Supplementary information [file 41598_2019_40962_MOESM1_ESM.pdf]

Epidemiological evaluation of Latvian control measures for African swine fever in wild boar on the basis of surveillance data

Katja Schulz, Edvīns Oļševskis, Christoph Staubach, Kristīne Lamberga, Mārtiņš Seržants, Svetlana Cvetkova, Franz Josef Conraths and Carola Sauter-Louis

Table S1: Number of ASFV genome-positive and -negative samples, samples originating from animals found dead or hunted and samples from hunted wild boar younger than one year or or older. Samples with inconclusive information for one of the variables (age, carcass or ASFV genome test result) were excluded from analyses. Highlighted lines indicate the period, in which the measures were applied.

Measure	Time period	ASFV genome			Carcass type		Age of hunted wild boar	
		positive	negative	Estimated ASFV genome prevalence	found dead	hunted	< 1 year	> 1 year
Incentives to all persons who report dead wild boar to the veterinary authorities (M1)	Measure period	206	2,359	0.08	219	2,346	1,085	1,480
	Control period	360	4,313	0.08	300	4,373	1,581	3,092
Incentives to hunters for hunted wild boar (M2)	Measure period	79	958	0.08	89	948	282	755
	Control period	243	1,900	0.11	241	1,902	545	1,598
Restrictions on driven hunts (M3a)	Measure period	104	1,328	0.07	104	1,328	752	680
	Control period	28	1,228	0.02	24	1,232	450	806
Restrictions on driven hunts (M3b)	Measure period	110	2,223	0.05	50	2,283	988	1,345
	Control period	28	1,228	0.02	24	1,232	450	806
Incentives for hunting adult and sub-adult female wild boar (M4a)	Measure period	100	2,019	0.05	51	2,068	879	1,240
	Control period	105	1,246	0.08	110	1,241	715	636
Incentives for hunting adult and sub-adult female wild boar (M4b)	Measure period	53	2,356	0.02	49	2,360	607	1,802
	Control period	498	4,790	0.09	493	4,795	1,956	3,332
Permission to use sound moderators (silencers) and night vision devices for wild boar hunting (M5)	Measure period	360	4,313	0.08	300	4,373	1,581	3,092
	Control period	206	2,359	0.08	219	2,346	1,085	1,480

Table S2: Results of the model analyses for Measure 1 (Incentives to all persons who report dead wild boar to the veterinary authorities) using a multivariable Bayesian regression model. The final result (highlighted) was chosen on the basis of the model performance (Deviance Information Criterion (DIC) and Effective Number of Parameters (pD)). *Mean/Std.Dev. >1.96, indicating statistical significance.

**Measure 1**	**Model**	**Mean**	**SD**	**Median (95% BCI)**	**Mean/St.Dev.***	**DIC**	**pD**
Proportion of wild boar found dead	M1	1.62	0.84	1.57 (0.06- 3.32)	1.93	3505.08	14.97
	M1	1.31	0.91	0.59 (-4.06 - 7.52)	1.45	3477.05	16.17
	Age	0.54	0.10	3.79 (-4.05 - 9.00)	5.65		
	M1	0.47	0.61	0.43 (-0.73 - 1.73)	0.77	3503.60	13.42
	Prevalence	6.16	1.74	6.05 (2.81 - 9.77)	3.54		
	M1	0.49	0.68	0.48 (-4.02 - 8.60)	0.72	3474.60	14.40
	Age	0.53	0.10	0.52 (-4.00 - 8.36)	5.49		
	Prevalence	6.01	1.88	3.80 (-3.99 - 9.23)	3.20		
Estimated ASFV genome prevalence	M1	0.87	0.67	-0.29 (- 4.07 - 5.54)	1.31	3867.56	14.67
	M1	0.81	0.68	-0.30 (-2.75 - 1.56)	1.19	3795.78	15.77
	Age	0.79	0.09	5.25 (-2.78 - 5.63)	8.73		
	M1	-0.47	0.56	-0.47 (-2.75 - 0.95)	0.84	1962.90	11.16
	Carcass type	5.32	0.15	5.30 (-2.78 - 5.65)	34.58		
	M1	-0.63	0.53	-0.48 (-3.96 - 5.50)	1.20	1921.28	11.59
	Age	0.94	0.14	0.56 (-3.96 - 5.52)	6.75		
	Carcass type	5.37	0.15	1.16 (-4.02 - 5.57)	35.97		

Table S3: Results of the model analyses for Measure 2 (Incentives to hunters for hunted wild boar) using a multivariable Bayesian regression model. The final result (highlighted) was chosen on the basis of the model performance (Deviance Information Criterion (DIC) and Effective Number of Parameters (pD)). *Mean/Std.Dev. >1.96, indicating statistical significance.

Measure 2	Model	Mean	SD	Median (95% BCI)	Mean/St.Dev.*	DIC	pD
Proportion of wild boar hunted	M2	0.64	0.57	0.80 (-2.01 - 13.57)	1.12	2108.67	5.42
	M2	0.65	0.79	0.68 (-0.83 - 1.88)	0.82	2055.60	6.52
	Age	-0.91	0.12	-0.91 (-1.14 - -0.67)	7.55		
	M2	0.22	2.89	0.68 (-1.07 - 2.48)	0.08	2107.98	5.98
	Prevalence	-1.14	33.13	-0.90 (-5.80 - 14.39)	0.03		
	M2	1.12	1.73	0.92 (-1.75 - 6.03)	0.65	2054.99	7.08
	Age	-0.91	0.12	-0.79 (-1.15 - 3.79)	7.67		
	Prevalence	9.48	21.48	1.08 (-10.76 - 39.68)	0.44		

Table S4: Results of the model analyses for Measure 3a (Restrictions on driven hunts) using a multivariable Bayesian regression model. The final result (highlighted) was chosen on the basis of the model performance (Deviance Information Criterion (DIC) and Effective Number of Parameters (pD)). *Mean/Std.Dev. >1.96, indicating statistical significance.

Measure 3a	Model	Mean	SD	Median (95% BCI)	Mean/St.Dev.*	DIC	pD
Estimated ASFV genome prevalence	M3a	0.21	1.12	0.29 (-1.94 - 1.24)	0.19	1001.47	8.83
	M3a	0.06	1.20	0.34 (-5.19 - 6.50)	0.05	1000.63	9.95
	Age	0.31	0.18	0.27 (-5.27 - 6.63)	1.70		
	M3a	0.41	0.80	0.21 (-5.19 - 6.49)	0.51	424.18	5.28
	Carcass type	6.00	0.32	0.53 (-5.26 - 6.62)	19.02		
	M3a	0.52	0.84	0.48 (-2.99 - 1.09)	0.61	422.36	6.26
	Age	0.64	0.32	0.63 (1.05 - 1.26)	1.99		
	Carcass type	6.15	0.33	6.13 (-0.91 - 6.83)	18.80		

Table S5: Results of the model analyses for Measure 3b (Restrictions on driven hunts) using a multivariable Bayesian regression model. The final result (highlighted) was chosen on the basis of the model performance (Deviance Information Criterion (DIC) and Effective Number of Parameters (pD)). *Mean/Std.Dev. >1.96, indicating statistical significance.

Measure 3b	Model	Mean	SD	Median (95% BCI)	Mean/St.Dev.*	DIC	pD
Estimated ASFV genome prevalence	M3b	0.74	0.70	0.55 (-0.87 - 1.84)	1.06	1159.86	6.38
	M3b	0.73	0.71	0.77 (-0.87 - 2.06)	1.03	1161.66	7.51
	Age	0.01	0.18	0.00 (-0.37 - 0.35)	0.04		
	M3b	0.49	0.85	0.41 (-4.98 -5.97)	0.57	818.04	6.44
	Carcass type	5.41	0.34	0.46 (-5.06 - 5.93)	15.98		
	M3b	0.47	0.81	0.61 (-1.43 - 2.01)	0.58	816.20	7.14
	Age	0.43	0.23	0.46 (0.05 - 1.43)	1.91		
	Carcass type	5.54	0.34	5.46 (-4.15 - 6.19)	16.26		

Table S6: Results of the model analyses for Measure 4a (Incentives for hunting adult and sub-adult female wild boar) using a multivariable Bayesian regression model. The final result (highlighted) was chosen on the basis of the model performance (Deviance Information Criterion (DIC) and Effective Number of Parameters (pD)). *Mean/Std.Dev. >1.96, indicating statistical significance.

Measure 4a	Model	Mean	SD	Median (95% BCI)	Mean/St.Dev.*	DIC	pD
Proportion of wild boar hunted	M4a	3.00	1.40		2.14	1191.6	9.49
	M4a	2.94	1.27	0.00 (-2.79 - 4.18)	2.32	1193.50	10.48
	Age	-0.91	0.12	-0.91 (1.14 - -0.67)	7.55		
	M4a	0.09	1.04	0 (-1.96 - 3.23)	0.09	1191.49	7.40
	Prevalence	-12.62	2.88	-0.24 (-16.57 - 3.15)	4.38		
	M4a	0.19	0.89	0 (-11.41 - 3.77)	0.21	1193.36	8.18
	Age	0.03	0.16	0 (-11.10 - 3.70)	0.19		
	Prevalence	-12.41	2.62	0 (-14.96 - 3.99)	4.73		

Table S7: Results of the model analyses for Measure 4b (Incentives for hunting adult and sub-adult female wild boar) using a multivariable Bayesian regression model. The final result (highlighted) was chosen on the basis of the model performance (Deviance Information Criterion (DIC) and Effective Number of Parameters (pD)). *Mean/Std.Dev. >1.96, indicating statistical significance.

Measure 4b	Model	Mean	SD	Median (95% BCI)	Mean/St.Dev.*	DIC	pD
Proportion of wild boar hunted	M4b	- 0.60	1.27	0.35 (-13.29 - 3.91)	0.47	3658.14	18.31
	M4b	- 0.37	1.15	0.41 (-1.05 - 3.07)	0.33	3653.90	18.67
	Age	- 0.23	0.10	-11.03 (-13.79 - 2.94)	2.27		
	M4b	0.39	0.46	0.43 (-0.74 - 3.07)	0.84	3656.79	9.33
	Prevalence	- 11.26	1.40	-11.14 (-13.88 - 2.94)	8.01		
	M4a	0.39	0.50	0.41 (-12.98 - 3.87)	0.78	3653.51	10.96
	Age	- 0.21	0.10	-0.06 (-12.65 - 3.93)	2.16		
	Prevalence	- 11.11	1.39	-0.56 (-13.50 - 3.92)	7.97		

Table S8: Results of the model analyses for Measure 5 (Permission to use sound moderators (silencers) and night vision devices for wild boar hunting) using a multivariable Bayesian regression model. The final result (highlighted) was chosen on the basis of the model performance (Deviance Information Criterion (DIC) and Effective Number of Parameters (pD)). *Mean/Std.Dev. >1.96, indicating statistical significance.

Measure 5	Model	Mean	SD	Median (95% BCI)	Mean/St.Dev.*	DIC	pD
Proportion of wild boar hunted	M5	1.37	0.84	1.23 (-3.26 - 3.15)	1.63	3504.91	14.71
	M5	1.40	0.82	1.23 (-0.22 - 2.97)	1.71	3476.86	16.06
	Age	-0.53	0.10	-0.72 (-4.02 - 2.50)	5.37		
	M5	1.07	0.79	1.21 (-0.29 - 2.96)	1.35	3508.87	15.02
	Prevalence	-1.72	1.34	-1.23 (-4.17 - 2.52)	1.28		
	M5	1.08	0.83	1.13 (-3.16 - 3.06)	1.30	3480.30	16.53
	Age	- 0.53	0.10	0.73 (-2.88 - 3.05)	5.33		
	Prevalence	- 0.94	1.47	0.78 (-3.55 - 3.13)	0.64		
Estimated ASFV genome prevalence	M5	-0.95	0.71	-0.56 (-5.46 - 1.59)	1.34	3867.62	14.69
	M5	-0.73	0.70	0.08 (-2.46 - 1.53)	1.05	3796.37	15.87
	Age	0.79	0.09	-5.17 (-5.59 - 0.83)	8.59		
	M5	0.50	0.58	0.19 (-2.46 - 1.61)	0.85	1962.70	11.16
	Carcass type	-5.32	0.15	-5.22 (-5.59 - -0.22)	34.62		
	M5	0.64	0.49	0.27 (-5.44 - 1.71)	1.30	1920.90	11.09
	Age	0.94	0.14	0.52 (-5.46 - 1.58)	6.84		
	Carcass type	- 5.36	0.16	-1.11 (-5.58 - 1.54)	34.39		

Model description (multivariable Bayesian regression model) adapted from Staubach, et al. [1].

The parameter π_i constitutes either

a) The probability that a wild boar sample *i* is positive for ASFV genome depending on the time *t,* the age (α) and the origin of sample (carcass) (β).

or

b) The probability that a wild boar *i* is found dead depending on the time *t*, the age (α) and the estimated ASFV prevalence (β) per month in the study area.

The parameter π_i is modeled with a logistic model

$$\log\left(\frac{\pi_i}{1-\pi_i}\right) = \mu + x + \alpha + \beta + \varphi_t$$

μ = intercept

x = fixed effect for the control measure

α = fixed effect for age

β = fixed effect for carcass type (a) or ASFV genome prevalence (b)

φ_t = time effect on the time *t*

The time effect is modelled as a special case of Random Walk (RW) and *a priori* normal-distributed

$$\varphi_t \sim N(2\varphi_{t-1} - \varphi_{t-2} / \sigma_\varphi^2)$$

For the unknown variance parameter σ^2_φ, we used an inverse Gamma prior $\sigma^2_\varphi \sim$ Inv-gamma (1, 0.05).

1 Staubach, C. *et al.* Bayesian space-time analysis of Echinococcus multilocularis-infections in foxes. *Veterinary Parasitology* **179**, 77-83, doi:10.1016/j.vetpar.2011.01.065 (2011).
